# Low Complexity Mode Decision for 3D-HEVC

**DOI:** 10.1155/2014/392505

**Published:** 2014-08-28

**Authors:** Qiuwen Zhang, Nana Li, Yong Gan

**Affiliations:** College of Computer and Communication Engineering, Zhengzhou University of Light Industry, Zhengzhou 450002, China

## Abstract

High efficiency video coding- (HEVC-) based 3D video coding (3D-HEVC) developed by joint collaborative team on 3D video coding (JCT-3V) for multiview video and depth map is an extension of HEVC standard. In the test model of 3D-HEVC, variable coding unit (CU) size decision and disparity estimation (DE) are introduced to achieve the highest coding efficiency with the cost of very high computational complexity. In this paper, a fast mode decision algorithm based on variable size CU and DE is proposed to reduce 3D-HEVC computational complexity. The basic idea of the method is to utilize the correlations between depth map and motion activity in prediction mode where variable size CU and DE are needed, and only in these regions variable size CU and DE are enabled. Experimental results show that the proposed algorithm can save about 43% average computational complexity of 3D-HEVC while maintaining almost the same rate-distortion (RD) performance.

## 1. Introduction

With the development of the technology of 3D television (3DTV) and free viewpoint television (FTV), 3D video coding attracts more and more attention. The typical 3D video is represented using the multiview video plus depth (MVD) format [1], in which few captured texture videos as well as associated depth maps are used. The depth maps provide per-pixel with depth corresponding to the texture video that can be used to render arbitrary virtual views by using depth image based rendering (DIBR) [2, 3]. In recent years, high efficiency video coding- (HEVC-) based 3D video coding (3D-HEVC) technology [4, 5] is now being standardized by joint collaborative team on 3D video coding (JCT-3V) as an extension to HEVC [6, 7]. From the JCT-3V meetings, the developed coding schemes for 3D-HEVC mainly use HEVC together with exploiting temporal and interview correlation. Thus, many coding tools applied in 3D-HEVC are based on the hybrid coding scheme and highly related to HEVC. Different from single-view video coding, 3D-HEVC uses disparity estimation (DE) to reduce the interview redundancy. The test model of 3D-HEVC uses the variable size coding unit (CU) and DE to exploit both temporal and view correlation within temporally successive pictures and neighboring views. This technique achieves the highest possible coding efficiency, but it results in extremely large encoding time which obstructs it from practical use. Therefore, it is necessary to develop a method that can reduce complexity of 3D-HEVC with minimal loss of image quality. Fast CU size and DE decision algorithms for encoding multiview video plus depth are extremely necessary.

Recently, a number of efforts have been proposed to reduce the computational complexity for the HEVC encoder. An effective CU size decision method is proposed in [8] to reduce encoding complexity of HEVC. Besides, a fast mode decision method is proposed based on the direction information of the spatially adjacent CUs in [9]. Fast intramode decision method [10] uses edge information of the current prediction unit (PU) to choose a reduced set of candidate prediction directions. A complexity control method [11] is proposed based on fast mode decision algorithm that dynamically adjusts the depth of the CU defined by quadtree-based structures. A fast CU splitting and pruning method [12] is presented based on early CU split and pruning decision for HEVC intracoding. All these methods are efficient in reducing computational complexity with small degradation in coding performance. However, these methods are not directly applicable to the 3D-HEVC, where high computational complexity is intrinsically related to the use of new prediction coding structures for depth-enhanced multiview formats.

To the best of our knowledge, studies devoted to investigation of complexity reduction of the 3D-HEVC are rarely found in the literatures. To this end, this paper proposes a fast CU size and DE mode decision algorithm to reduce 3D-HEVC computational complexity. Our algorithm exploits the correlation between depth map and motion activity to reduce the 3D-HEVC computational complexity. Since the depth maps can efficiently describe the actual 3D information, the potential of utilizing depth map for fast CU size and DE algorithm is promising. The proposed algorithm consists of two approaches: fast CU size decision and selective disparity estimation. Based on these two criteria, the number of candidate modes in a view is reduced, the disparity search is selectively enabled, and the search range of CU size is adaptively determined. Experimental results demonstrate that the proposed algorithm can significantly reduce the computational complexity of 3D-HEVC while maintaining almost the same rate-distortion (RD) performance as the original encoder.

The rest of the paper is organized as follows. In Section 2, we analyze the correlation of depth maps among motion activities and propose a fast CU size and DE algorithm based on depth map. Experimental results and conclusions are given in Sections 3 and 4, respectively.

## 2. Low Complexity Mode Decision Algorithm

### 2.1. Fast CU Size Decision Based on Depth Map

**Figure 1:**
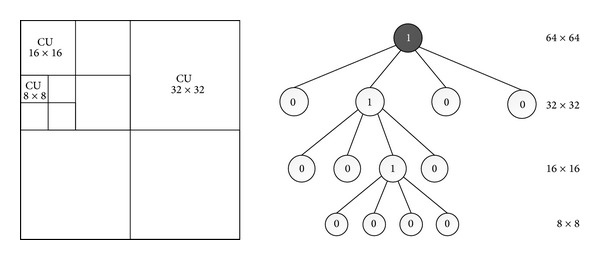
The simple example for recursive CU.

3D-HEVC inherits an advanced quadtree-based coding approach from HEVC, wherein a picture is divided into coding tree units (CTUs) [13]. Those are equivalent to macroblocks (MBs) in previous video coding standards such as H.264/AVC. The CTU can then be split into four CUs, and the CU is the basic unit of region splitting used for inter-/intraprediction, which allows recursive subdividing into four equally sized blocks. This process gives a content-adaptive coding tree structure comprised of CU blocks that may be as large as a treeblock or as small as 8 × 8 pixels.  Figure 1 shows the architecture of tree structured CUs. A specified maximum depth level is set to limit the CU split recursion. At each depth level (CU size), 3D-HEVC performs motion estimation (ME) with different sizes including 2*N* × 2*N*, 2*N* × *N*, *N* × 2*N*, and *N* × *N*. Similar to the joint model of HEVC, the mode decision process in 3D-HEVC is performed using all the possible CU sizes (depth levels) and prediction modes to find the one with the least RD cost using Lagrange multiplier [8]. This achieves the highest coding efficiency but requires a very high computational complexity. In this paper, we propose a fast CU size decision algorithm for 3D-HEVC. Since the optimal depth level is highly content-dependent, it is not efficient to use all levels. We can determine CU depth range (including the minimum depth level and the maximum depth level) and skip some specific depth levels rarely used in the mode decision process.

3D-HEVC usually allows a maximum CU size that equals 64, and the depth level range is from 0 to 3. The CU depth level has a fixed range for a whole video sequence in 3D-HEVC reference software [15]. In fact, small depth level values tend to be chosen for CUs in small global motion or homogeneous texture region, and large depth level values are chosen for CUs with large global motion or rich texture region. We can see from experiments of 3D-HEVC coding that the depth value of “0” occurs very frequently for large homogeneous texture region. On the other hand, the depth value of “0” is rarely chosen for treeblocks with active motion or rich texture region. These results show that CU depth level range should be adaptively determined based on the motion and texture property of treeblocks. In 3D video coding, a depth map represents a relative distance from a camera to an object in the 3D space, it can be regarded as a grayscale image using dark and bright values to represent far and close object, and the object depth not only represents the physical object position in 3D space but also indicates the motion activity of the object itself on the image plane. Under the condition that cameras are set up in a close parallelized structure, the depth maps are correlated to the texture video motion fields. Consequently, the optimal depth value level of current treeblock may have a strong correlation with its associated depth maps. Based on this concept, we can make use of depth map and motion activity correlations to analyze region properties and skip ME on unnecessary CU sizes.

In 3D space, the motion of the close object in depth map is usually higher than that of the far object in depth map [16]; the major interesting ratio of the video object should be put in the middle region. To achieve great saving in coding time while minimizing the loss in coding efficiency, the depth level of a treeblock having limited contribution to coding efficiency should be skipped. So we use depth information to filter out the unsuitable mode candidates to speed up the encoding process. Since the depth map indicates the physical position of the object in the 3D space, the potential of utilizing depth map for fast CU size decision is promising. In a nature video test sequence, the degree of motion activity for the object with near region from the 3D space will usually be higher than that of the object with far region. Based on this observation, the depth map can be used to classify the motion activities of video objects by this property. Thus, we first classify the objects motion activities into three regions: near region, middle region, and far region according to the depth map value. Near region represents a medium local motion or a smooth texture on the 3D space plane, middle region represents a large global motion or rich texture on the 3D space plane, and far region represents a small global motion or a homogeneous texture on the 3D space plane. Considering that the optimal CU depth level is highly dependent on object motion activities and texture characteristic mentioned above (small depth level values are suitable for CUs in small global motion or homogeneous texture region, and large depth level values are reasonable for CUs with large global motion or rich texture region), we can establish a relationship between the depth level and the depth map. By utilizing the depth map, we can classify scenes according to the corresponding depth map value and assign the suitable CU depth level candidates. Based on the aforementioned analysis, the 3D video space is classified into three regions based on the depth information: near region, middle region, and far region; the treeblocks classification based on depth map can be represented by the following equations:

(1) Ztreeblock≤Z0treeblock∈Near  region  mode,Z0<Ztreeblock≤Z1treeblock∈Middle  region  mode,Ztreeblock>Z1treeblock∈Far  region  mode,

where *Z*_treeblock_ is the depth map value of current treeblocks and *Z*_0_ and *Z*_1_ are chosen based on the sequence characteristics and set to 0 and 255, respectively. The selection of the thresholds *Z*_0_ and *Z*_1_ should greatly reduce the 3D-HEVC computational complexity while keeping a high accuracy in CU size decision. Based on extensive experiments, the thresholds *Z*_0_ and *Z*_1_ are, respectively, set to 200 and 30, which achieve a good and consistent performance on a variety of test sequences with different texture characteristics and motion activities.

Extensive simulations have been conducted on 8 video sequences with different resolutions to analyze the depth level distribution for these three types of treeblocks. Among these test sequences,* Kendo*,* Balloons*, and* Newspaper* are in 1024 × 768 resolution, while* Undo_Dancer*,* GT_Fly*,* Poznan_Street*,* Poznan_Hall2*, and* Shark* are in 1920 × 1088 resolution. The test conditions are as follows: there is I-B-P view structure, there is test of 200 frames for each sequence, quantization parameter (QP) is chosen with 26, 31, 36, and 41, group of pictures (GOP) size is 8, treeblock size is 64, and context-adaptive binary arithmetic coding (CABAC) is used for entropy coding. By exploiting the exhaustive intramode decision in HTM under the aforementioned test conditions, we investigate the depth level distribution for these three types of treeblocks.

**Figure 2:**
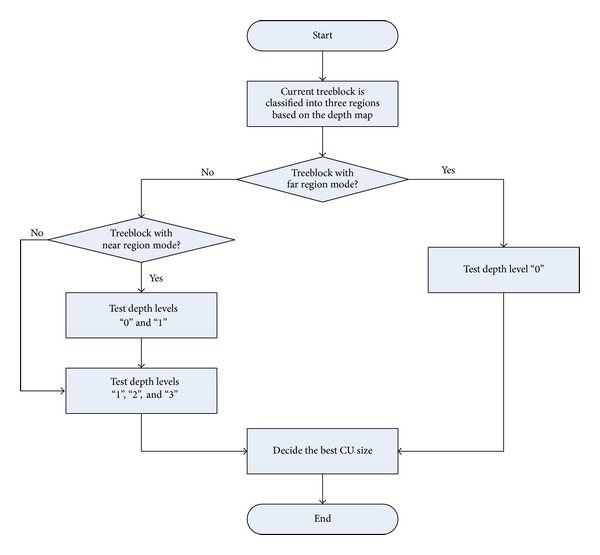
Flowchart of the proposed fast CU size decision algorithm.

Table 1 shows the depth level distribution for each type of treeblocks, where “level 0,” “level 1,” “level 2,” and “level 3” are the depth levels of treeblocks. It can be seen that, for treeblocks with near region mode, about 70% of total treeblocks choose the optimal depth level with “0” and about 21% treeblocks choose the optimal depth value with “1.” In other words, if the maximum depth level is set to be “1,” it will most likely cover about 91% of treeblocks. For treeblocks with middle region mode, about 96% of treeblocks choose depth levels with “1,” “2,” and “3.” If the minimum depth level is set to be “1” and the maximum depth level is set to be “2,” it will most likely cover about 96% of treeblocks. On the other side, the probability of choosing the depth level with “0” is very low, less than 4%, and thus intraprediction on depth level of “0” (CU size 64 × 64) can be skipped. For treeblocks with far region mode, the probability of choosing the depth levels of “0” is more than 90%, and thus intraprediction on depth levels of “1,” “2,” and “3” (CU sizes 32 × 32, 16 × 16, and 8 × 8) can be skipped. Based on the above analysis, the candidate depth levels that will be tested using RD optimization (RDO) for each treeblock are summarized in Table 2. With the proposed fast CU size decision, most of treeblocks can skip one to three tested depth levels. A flowchart of the proposed fast CU size decision is given in Figure 2.

### 2.2. Selective Disparity Estimation Based on Depth Map

One of the most important aspects for efficient MVD coding is the redundancy reduction among different views at the same time instance, for which the content is usually rather similar and only varies by a slightly different viewing position. As a coding tool for dependent views, the concept of disparity estimation has been involved as an alternative to motion estimation in 3D-HEVC encoders. Here, ME refers to interpicture prediction that uses already coded pictures of the same view at different time instances, while DE refers to interpicture prediction that uses already coded pictures of other views at the same time instance [17]. DE is also used in the multiview video coding (MVC) extension of H.264/MPEG-4 AVC and similarly the coding treeblock syntax and decoding process of HEVC remain unchanged when adding DE to 3D-HEVC codec. Only the high-level syntax has been modified so that already coded video pictures of the same access unit can be inserted into the reference picture lists [1]. Thus, in the joint mode of 3D-HEVC, both ME and DE are included in the encoding process. This achieves the highest coding efficiency but requires a very high computational complexity. Disparity estimation is to search the best matched block in frames from neighbor views. As mentioned above, disparity prediction is used to exploit interview dependence. Although temporal prediction is generally the most efficient prediction mode in 3D-HEVC, it is sometime necessary to use both DE and ME rather than only use ME to achieve better predictions. In general, temporal motion cannot be characterized adequately, especially for regions with nonrigid motion and regions with motion boundaries. For the former, ME based on simple translation movement usually fails and thus produces a poor prediction. For the latter, regions with motion boundaries are usually predicted using small mode sizes with larger magnitude of motion vectors and higher residual energy. As mentioned above, the depth map indicates the motion activity of the object itself. Normally, areas with homogeneous motion probably belong to the far depth region, and areas with complex motion probably belong to the middle depth region. Since, for a normal parallelized camera setting, the major object motion should be put in the middle region, thus, the regions with far depth region are more likely to choose temporal prediction, and regions with middle depth region are more likely to choose interview prediction.

**Figure 3:**
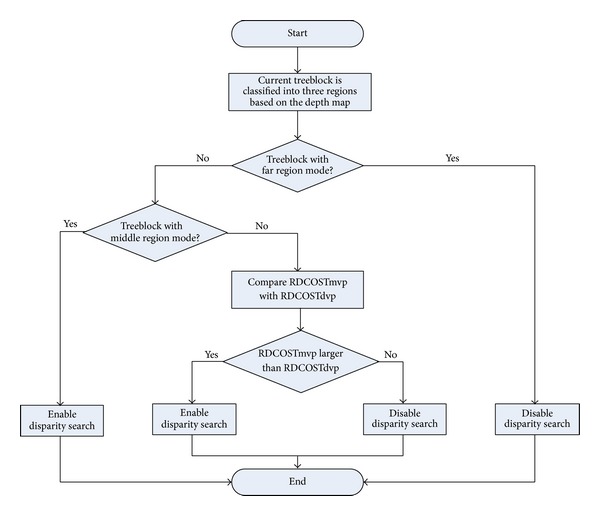
Flowchart of the proposed selective disparity estimation algorithm.

Table 3 shows probabilities of choosing interview prediction and temporal prediction for each type of treeblocks classified based on depth map. For treeblocks with near region mode, the average probabilities of choosing temporal prediction and interview prediction are 87% and 13%, respectively. For treeblocks with middle region, they are 69% and 31%. For treeblocks with far region mode, they are 97% and 3%. We can see from Table 3 that treeblocks with far region mode are much more likely to choose temporal prediction. Thus, for far region mode treeblocks, the procedure of the interview prediction can be skipped with only a very low miss detection ratio, by using the optimal prediction mode chosen by the full interview and temporal prediction modes. But, for middle region mode treeblocks (treeblocks with near region mode), the average probabilities of choosing interview prediction are 31% (13%). Although the test sequences such as “Poznan_Hall2” and “Newspaper” contain large area of the homogeneous textures and low-activity motion, which are more likely to be encoded with temporal prediction, the probability of interview prediction for treeblock with middle region and near region is still the highest. Thus, if we disable interview prediction in middle region and near region, the coding efficiency loss is not negligible. Based on the aforementioned analysis, we propose a selective disparity estimation algorithm in which disparity search is selectively enabled. For treeblocks with far region mode, disparity search is skipped, while, for treeblocks with middle region mode, disparity search is enabled. For treeblocks with near region mode, the RD cost of the motion vector predictor (MVP) is compared with that of the disparity vector predictor (DVP). If the RD cost of MVP is larger than that of DVP, disparity search is enabled; otherwise it is disabled. A flowchart of the scheme is given in Figure 3.

## 3. Experimental Results

In order to evaluate the performance of the proposed fast algorithms, the fast CU size decision and selective disparity estimation algorithms are implemented on the recent 3D-HEVC reference software (3DV-HTM 4.1) [18]. All the simulations are defined under the common test conditions (CTC) [19] defined by JCT-3V. We have tested the proposed algorithms on eight sequences defined in the CTC with two resolutions (1024 × 768 and 1920 × 1088). The encoder configuration is as follows: there are 3 view cases, the GOP length is 8 with an intraperiod of 24, HEVC codecs are configured with 8-bit internal processing, the coding treeblock has a fixed size of 64 × 64 pixels and a maximum CU depth level of 4, resulting in a minimum CU size of 8 × 8 pixels, and CABAC is used as the entropy coder. The proposed algorithm is evaluated with QP combinations for texture video and depth map (25, 34), (30, 39), (35, 42), and (40, 45). The experiments test 200 frames for each sequence. Each sequence is composed of three texture videos and three depth map views: the center-the left-the right views (in coding order). After encoding, the intermediate rendered views were synthesized between all views. The intermediate rendered views are generated at the receiver using view synthesis reference software (VSRS) algorithm provided by MPEG [20].

### 3.1. Individual Performance Results of the Proposed Algorithm

Table 4 gives the individual evaluation results of the proposed algorithm compared with the original 3DV-HTM algorithm, that is, fast CU size decision (FCUS) and selective disparity estimation (SDE), respectively. The Bjontegaard delta PSNR (BDPSNR) [21] represents the average PSNR gain, bitrate (BDBR) represents the improvement of total bitrates for 3D video coding, and “Dtime (%)” represents the entire coding time change in percentage. The “texture” represents average PSNR for coded texture video views. The “rendered” represents average PSNR for rendered views. Rendered PSNR on rendered view distortion can be measured by comparing the coded rendered view with the image rendered with uncompressed texture videos and depth map [22]. The bitrate under consideration is the sum of the bitrates of the three coded texture videos and depth map views.

The proposed two approaches can greatly reduce the coding time with similar coding efficiency for all sequences. FCUS can save about 35% coding time over all sequences. The coding efficiency loss is very negligible with 0.02 dB–0.08 dB PSNR drop for average texture videos and 0.01 dB–0.04 dB PSNR drop for average rendered views. This result indicates that FCUS can efficiently skip unnecessary depth levels in CU size decision. As far as the SDE algorithm is concerned, 13% coding time has been reduced. And, for average texture videos coding, the average PSNR drop is 0.03 dB, or the bitrate increases about 0.84% on average. For average rendered views coding, the average PSNR drop is 0.015 dB, or the increase of bitrate is about 0.24% on average, which is negligible. The foregoing result analysis indicates that SDE can efficiently reduce the coding time while maintaining almost the same coding performance as the 3D-HEVC encoder.

### 3.2. Combined Results

In the following, we will analyze the experimental result of the proposed overall algorithm, which incorporates FCUS and SDE. The comparison results of the overall algorithm are shown in Table 5. The proposed overall algorithm can greatly reduce coding time for all sequences. It reduces 43% coding time under average texture videos and average rendered views conditions and achieves the better gain in coding speed compared to FCUS and SDE. Also a consistent gain in coding speed for all test sequences with the lowest gain of 36% for “Poznan_Street” and the highest gain for 53% for “Poznan_Hall2” is shown. The computation reduction is particularly high because the exhaustive CU size decision procedures of a significant number of CUs are reasonably skipped, and disparity estimation procedures of a significant number of CUs are not processed by the 3D-HEVC encoder. On the other hand, the coding efficiency loss is negligible; specifically, for average texture videos coding, the average PSNR drop is 0.055 dB, and the increase of bitrate is about 1.65% on average. For average rendered views coding, the average PSNR drop is 0.039 dB, and the increase of bitrate is about 1.16% on average.

**Figure 4:**
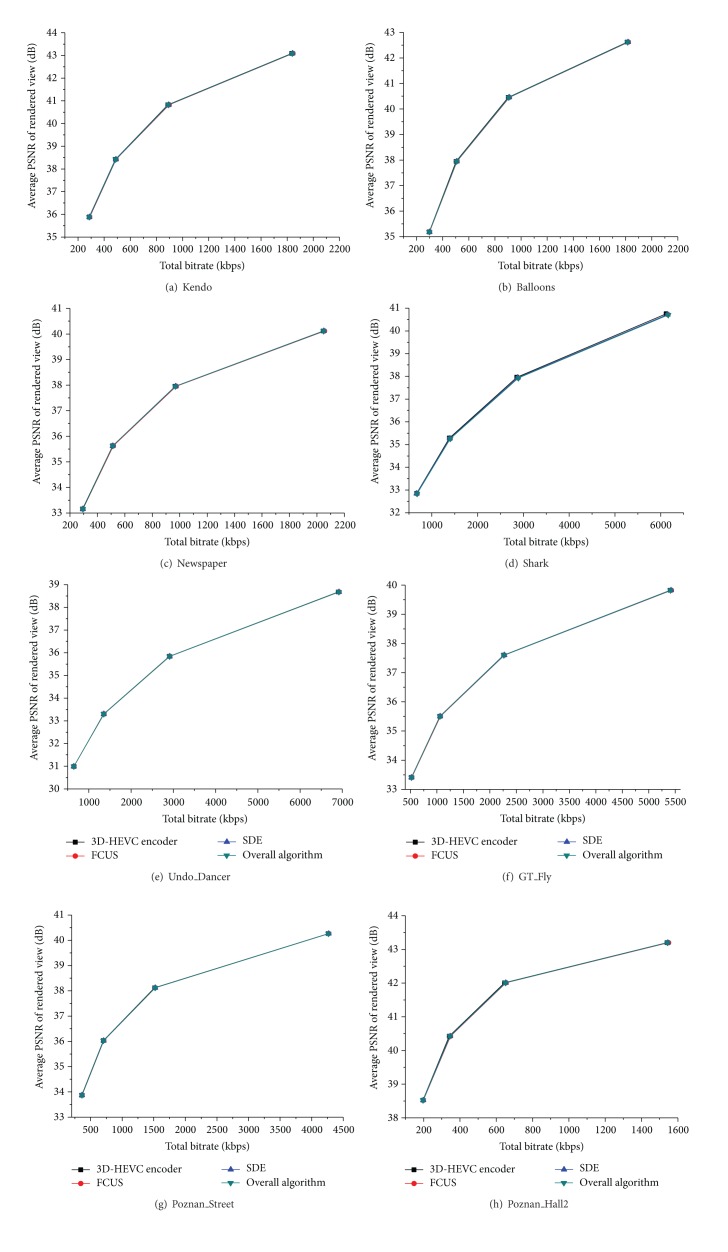
Rate-distortion curves comparison. The *x*-axis denotes total bitrate to code three texture videos and three depth maps. The *y*-axis denotes average PSNR of the rendered views.

Figure 4 gives RD curves of the proposed algorithms compared to 3D-HEVC, which are the total bitrates for the multiview texture video and depth map coding and average PSNR over all virtual views. As shown in Figure 4, the proposed algorithms (FCUS and SDE and the overall algorithm) perform almost the same coding efficiency from low to high bitrate compared to 3D-HEVC. Therefore, the proposed algorithm can efficiently reduce coding time while keeping nearly the same RD performance as 3D-HEVC.

### 3.3. Results of the Proposed Overall Algorithm Comparison with the State-of-the-Art Fast Algorithm

The comparison results of the overall algorithm and a state-of-the-art fast algorithm (content-adaptive complexity reduction scheme, CACRS [14]) are given in Table 6. Experimental results shown in Table 6 indicate that the proposed overall algorithm consistently outperforms CACRS. The proposed overall algorithm can save 12% encoding time on average compared to CACRS, with the lowest gain of 3% for “Undo_Dancer” and the highest gain of 22% for “Poznan_Hall2.” Additionally, the proposed overall algorithm achieves a better coding performance. For average texture videos coding, with 0.14 dB PSNR increases or 1.96% bitrate decreases compared to CACRS. For average rendered views coding, with 0.12 dB PSNR increases or 1.61% bitrate decreases compared to CACRS. Therefore, the proposed overall algorithm is more efficient than CACRS with better time saving and fewer bits. The above experimental results indicate that the proposed overall algorithm is efficient for all test sequences and consistently outperforms the recent fast algorithm for 3D-HEVC.

## 4. Conclusion

In this paper, we propose a low complexity mode decision algorithm to reduce the computational complexity of the 3D-HEVC encoder, which includes two fast approaches: fast CU size decision approach and selective disparity estimation approach. The recent 3D-HEVC reference software 3DV-HTM is applied to evaluate the proposed algorithm. The comparative experimental results show that the proposed algorithm can significantly reduce the computational complexity of 3D-HEVC while maintaining almost the same RD performances.

## Figures and Tables

**Table 1:** Statistical analysis of depth level distribution for three treeblocks types.

Sequences	Treeblocks in near region mode				Treeblocks in middle region mode				Treeblocks in far region mode			
	Level 0 (%)	Level 1 (%)	Level 2 (%)	Level 3 (%)	Level 0 (%)	Level 1 (%)	Level 2 (%)	Level 3 (%)	Level 0 (%)	Level 1 (%)	Level 2 (%)	Level 3 (%)
Kendo	67.4	23.9	7.6	2.1	3.6	29.2	36.8	30.4	91.2	7.2	1.3	0.3
Balloons	75.6	18.2	5.8	0.4	4.6	31.9	37.2	26.3	89.3	6.3	3.2	1.2
Newspaper	58.9	29.4	8.6	3.1	2.1	26.8	37.7	33.4	92.4	6.1	1.2	0.3
Shark	70.2	19.5	7.5	2.8	1.9	23.9	36.1	38.1	93.7	4.9	1.3	0.1
Undo_Dancer	81.2	15.3	3.2	0.3	3.2	30.9	37.3	28.6	91.5	5.8	2.1	0.6
GT_Fly	70.4	21.8	6.2	1.6	4.1	37.2	29.5	29.2	87.2	8.2	3.6	1.0
Poznan_Street	65.6	24.3	7.5	2.6	2.8	27.2	32.9	37.1	92.8	4.6	1.7	0.9
Poznan_Hall2	72.4	19.3	5.9	2.6	4.5	36.8	34.4	24.3	85.1	9.4	3.7	1.8

Average	70.2	21.5	6.5	1.9	3.4	30.5	35.2	30.9	90.4	6.6	2.3	0.8

**Table 2:** Candidate depth levels for three treeblocks types.

Treeblock type	Candidate depth levels	Depth range, [Depth_min⁡_, Depth_max⁡_]
Near region mode	0, 1	[0,1]
Middle region mode	1, 2, 3	[1,2, 3]
Far region mode	0	[0,0]

**Table 3:** Analysis of view prediction and temporal prediction distributions for three treeblocks types.

Sequences	Treeblocks in near region mode		Treeblocks in middle region mode		Treeblocks in far region mode	
	T (%)	V (%)	T (%)	V (%)	T (%)	V (%)
Kendo	87.4	12.6	62.3	37.7	98.1	1.9
Balloons	82.9	17.1	71.2	29.8	96.3	3.7
Newspaper	91.2	8.8	76.5	23.5	99.5	0.5
Shark	83.6	16.4	72.1	27.9	97.4	2.6
Undo_Dancer	91.3	8.7	64.2	35.8	96.8	3.2
GT_Fly	87.7	12.3	62.3	37.7	94.3	5.7
Poznan_Street	85.6	14.4	67.6	32.4	96.1	3.9
Poznan_Hall2	90.2	9.8	77.3	22.7	94.9	5.1

Average	87.4	12.5	69.2	30.9	96.7	3.3

“T” and “V” represent temporal prediction and view prediction, respectively.

**Table 4:** Results of each individual algorithm compared to 3DV-HTM.

Sequences	Texture						Rendered					
	FCUS			SDE			FCUS			SDE		
	BDBR (%)	BDPSNR (dB)	Dtime (%)	BDBR (%)	BDPSNR (dB)	Dtime (%)	BDBR (%)	BDPSNR (dB)	Dtime (%)	BDBR (%)	BDPSNR (dB)	Dtime (%)
Kendo	1.23	−0.03	−38	0.82	−0.02	−12	0.22	−0.02	−38	0.12	−0.01	−12
Balloons	1.41	−0.05	−32	0.35	−0.01	−8	0.37	−0.04	−32	0.32	−0.00	−8
Newspaper	0.78	−0.02	−39	0.96	−0.04	−17	0.16	−0.03	−39	0.26	−0.02	−17
Shark	2.17	−0.06	−42	1.13	−0.02	−21	0.43	−0.02	−42	0.33	−0.01	−21
Undo_Dancer	0.89	−0.08	−29	0.47	−0.01	−6	0.19	−0.03	−29	0.21	−0.02	−6
GT_Fly	1.54	−0.02	−34	1.34	−0.05	−11	0.67	−0.04	−34	0.14	−0.01	−11
Poznan_Street	0.82	−0.07	−26	0.96	−0.06	−16	0.32	−0.01	−26	0.32	−0.03	−16
Poznan_Hall2	0.94	−0.04	−44	0.72	−0.03	−9	0.43	−0.01	−44	0.21	−0.02	−9

Average	1.22	−0.046	−35	0.84	−0.03	−13	0.35	−0.025	−35	0.24	−0.015	−13

**Table 5:** Results of the proposed overall algorithm compared with 3DV-HTM.

Sequences	Overall algorithm					
	Texture			Rendered		
	BDBR (%)	BDPSNR (dB)	Dtime (%)	BDBR (%)	BDPSNR (dB)	Dtime (%)
Kendo	1.92	−0.04	−45	1.21	−0.03	−45
Balloons	1.78	−0.06	−39	0.84	−0.02	−39
Newspaper	1.27	−0.03	−46	1.27	−0.04	−46
Shark	2.34	−0.06	−48	1.29	−0.03	−48
Undo_Dancer	1.12	−0.09	−37	0.58	−0.02	−37
GT_Fly	1.54	−0.03	−41	1.82	−0.06	−41
Poznan_Street	1.45	−0.08	−36	1.29	−0.07	−36
Poznan_Hall2	1.76	−0.05	−53	0.96	−0.04	−53

Average	1.65	−0.055	−43	1.16	−0.039	−43

**Table 6:** Comparing the proposed overall algorithm with a state-of-the-art fast algorithm in [14].

Sequences	Texture			Rendered		
	BDBR (%)	BDPSNR (dB)	Dtime (%)	BDBR (%)	BDPSNR (dB)	Dtime (%)
Kendo	1.35	−0.09	−12	1.12	−0.08	−12
Balloons	1.54	−0.12	−8	1.38	−0.11	−8
Newspaper	3.12	−0.19	−21	2.59	−0.16	−21
Shark	1.69	−0.14	−15	1.34	−0.11	−15
Undo_Dancer	2.71	−0.18	−3	2.16	−0.14	−3
GT_Fly	1.89	−0.15	−5	1.54	−0.12	−5
Poznan_Street	1.63	−0.13	−6	1.21	−0.09	−6
Poznan_Hall2	1.72	−0.14	−22	1.53	−0.12	−22

Average	1.96	−0.14	−12	1.61	−0.12	−12
